# The Genus *Petunia* (Solanaceae): Evolutionary Synthesis and Taxonomic Review

**DOI:** 10.3390/plants14101478

**Published:** 2025-05-15

**Authors:** Luana S. Soares, João R. Stehmann, Loreta B. Freitas

**Affiliations:** 1 Department of Genetics, Universidade Federal do Rio Grande do Sul, Porto Alegre 90509-900, Brazil; lusousa.soares11@gmail.com; 2 Department of Botany, Universidade Federal de Minas Gerais, Belo Horizonte 31270-901, Brazil

**Keywords:** *Petunia*, taxonomy, phylogeny, species delimitation, evolutionary relationships, Solanaceae

## Abstract

Many plant groups exhibit complex evolutionary processes, including hybridization, incomplete lineage sorting, and variable evolutionary rates, which make species delimitation challenging. Molecular data have been essential for studying such groups, including *Petunia*, where local adaptation, allopatric speciation, pollinator interactions, and hybridization shape diversity and population structure. In this study, we produced the first broadly inclusive phylogenetic tree of *Petunia* using high-throughput DNA sequence data generated by genome complexity reduction-based sequencing (DArT), and incorporating all currently accepted taxa. Additionally, we reviewed previously published phylogenetic and phylogeographic studies on these species to support the taxonomic revision. Phylogenetic analyses based on SNPs were largely congruent, revealing two well-supported clades divided by corolla tube length, consistent with previous studies. These clades likely originated and diversified during the Pleistocene. The phylogenetic trees provided strong support for taxonomic changes, resolving long-standing uncertainties. We recognize *P. axillaris*, *P. parodii*, and *P. subandina* as independent species, elevate *P. integrifolia* subsp. *depauperata* to *P. dichotoma* Sendtn., and resurrect *P. guarapuavensis*. Additionally, our results highlighted unsolved questions regarding the evolutionary history of the short corolla tube clade, suggesting the need for further investigation into its diversification and genetic structure.

## 1. Introduction

Identifying species is a long-standing challenge in biology [1]. Species are the basic units used to understand evolutionary relationships and describe biodiversity, which are essential steps for nature conservation. Intricate evolutionary processes characterize many plant groups, such as hybridization, incomplete lineage sorting, and saltatory evolutionary rates. These processes can make it more challenging to distinguish taxa in such groups through morphology and genetics [2].

A widely accepted assumption [1] states that species represent distinct genetic ancestor-descendant lineages interconnected by populations across time and/or space. Some of these lineages are characterized by persistence in time and space, with individuals sharing a common phenotype, ecological niche, or behavior; these would thus be considered species. Traditionally, the most commonly applied delimitation criterion is morphology, occasionally supplemented by other data types under an integrative taxonomic approach. These approaches systematically combine genetics with other sources of evidence, allowing for greater confidence and less subjectivity in species delimitation.

Molecular data have been crucial for studying and identifying species in many plant groups (e.g., [3,4]). Evolutionary studies, including population genomics, phylogeography, and biogeography, depend on correct species identification and delimitation [2]. The taxonomy of recently divergent species is particularly challenging due to the lack of clear diagnostic traits, and applying integrative taxonomy may be helpful in combining morphological and genetic features [5].

The genus *Petunia* Juss. (Solanaceae) is known worldwide for its commercial hybrid, *P.* × *atkinsiana* (Sweet) D.Don ex W.H.Baxter (usually cited as *P.* × *hybrida* (Hook.) Vilm.), a model vascular plant for molecular studies [6] and a popular bedding plant [7]. This charismatic group of herbaceous and annual plants currently encompasses 16 species [8,9,10]. Two species are divided into subspecies, totaling 19 taxa [8]. The most recent and inclusive phylogenetic analysis was published about a decade ago [11] and did not include the two most recently described species. In that work, species were distributed into two main clades, strongly supported by the corolla tube length, which were further subdivided based on the elevation where species occur.

Several works employing different molecular markers and analytical methods produced discordant tree topologies (e.g., [11,12,13,14,15]). Furthermore, some authors [8,12,16] disagree regarding the number and circumscription of taxa or their status as species or subspecies.

Over time, differences in habitat, geographic distribution, morphological traits, and genetic variability have led authors to suggest taxonomic revisions for several taxa (e.g., [17,18,19]). Based on similar evidence, others have grouped variable individuals within the same taxon (e.g., [20,21]).

The *Petunia* species have been widely studied, and different evolutionary patterns arose from these studies. Local adaptation [22], allopatric speciation [15], plant-pollinator interaction [23], and center-peripheral diversification [24,25] are some of the most frequent processes suggested to explain species diversity and population structure. Another motor for variation has been interspecific hybridization [19,26,27,28], which, in addition to generating hybrid zones with high genetic and morphological diversity, also impacts variability in parental populations [22,29].

In this study, we produced the first broadly inclusive phylogenetic tree of *Petunia* using high-throughput DNA sequence data generated by the DArT sequencing method, incorporating almost all currently accepted taxa. Additionally, we reviewed previously published phylogenetic and phylogeographic studies on these species to support the taxonomic revision. We aimed to address the following questions: (1) What is the phylogenetic position of the recently described taxa? (2) Can higher genomic coverage clarify the species relationships more effectively than other molecular markers? (3) Does the phylogenetic positioning contribute to the taxonomy of the genus?

## 2. Results

### 2.1. Dataset

The DArT sequencing produced a comprehensive dataset that included 18 of the 20 *Petunia* taxa and outgroups (*Calibrachoa spathulata* (L.B. Sm. and Downs) Stehmann and Semir, *C. caesia* (Sendtn.) Wijisman, *C. humilis* (R.E. Fr.) Stehmann and Semir, and *C. ovalifolia* (Miers) Stehmann and Semir) with a total of 49,157,383 reads (Supplementary Table S1). Individual read counts ranged from 405,928 to 2,389,837, averaging 1,003,212 reads per sample. After processing, we obtained 11,495 high-quality biallelic SNPs, allowing for 10% missing data. The number of reads per individual and the total number of SNPs were similar to those reported in other studies that utilized comparable techniques and *Petunia* species. Unfortunately, we lost the sequences of *P. axillaris* subsp. *parodii* (Steere) Cabrera and *P. occidentalis* R.E. Fr. because the individual libraries did not meet our sequence quality and quantity criteria.

### 2.2. Evolutionary Relationships

The multiple methods used for inferring the evolutionary relationships between *Petunia* species based on SNPs were mostly congruent, and none were fully discordant. SNAPP was the method that disagreed most with the others, with distance values ranging from 0.26 to 0.30 in concordance (Supplementary Figure S1).

**Figure 1:**
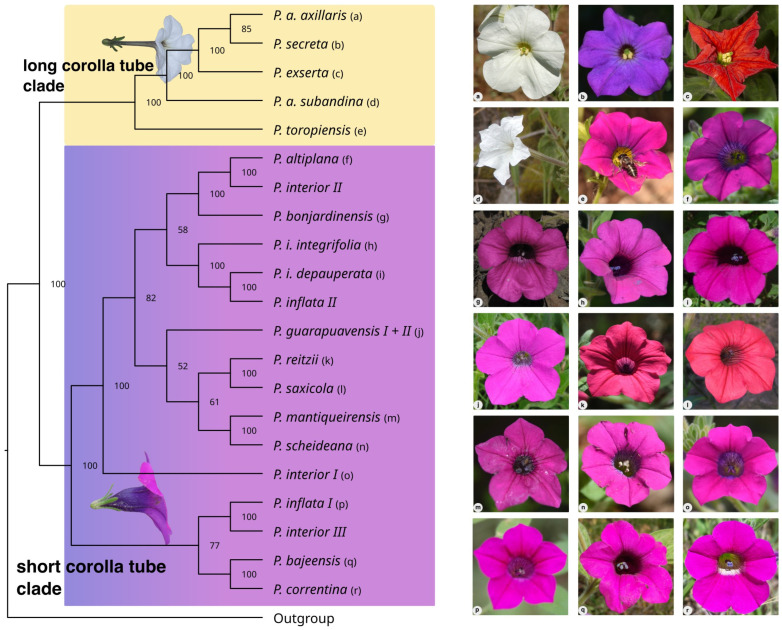
Best-scoring RAxML tree with bootstrap values at nodes. Clades are color-coded: yellow for long corolla tube species and purple for short corolla tube species. Photos of each species are shown on the right. Photo credits: Clemens Schlindwein, João Renato Stehmann, and Julian A. Greppi.

**Figure 2:**
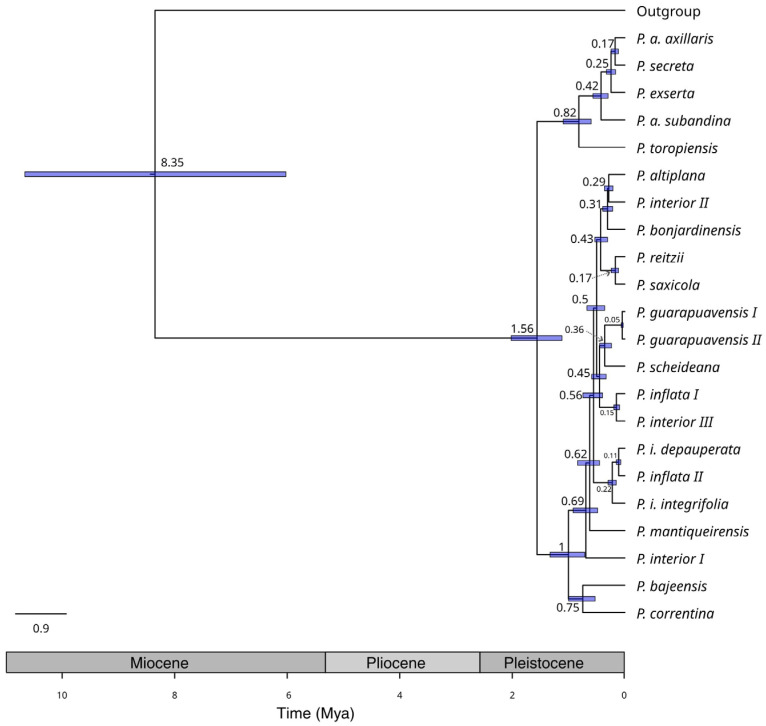
Evolutionary relationships among *Petunia* lineages inferred by SNAPP. The consensus tree is shown, with node ages and 95% confidence intervals (CIs) represented by node bars.

The species were divided into two main clades, as previous analyses based on diverse genetic markers indicated. These well-supported clades categorized species according to corolla tube length (Figure 1). The estimated origin time for these clades, based on SNPs, was approximately 1.5 million years ago (Mya; Figure 2), while species diversification began at around 1 Mya for the short corolla tube clade (ST) and roughly 0.8 Mya for the long corolla tube clade (LT).

In the LT, *P. axillaris* subsp. *axillaris* (Lam.) Britton, Sterns, Poggenb, and *P. secreta* Stehmann and Semir differ only by the corolla color, are grouped closer, and have *P. exserta* Stehmann as their sister species. These three species occur in the Pampa region, with *P. exserta* and *P. secreta* endemics, whereas *P. axillaris* subsp. *axillaris* is widely distributed in this ecosystem. *Petunia axillaris* subsp. *subandina* T. Ando, which occurs in the sub-Andean region in Argentina, appeared basal to the Pampean species and did not share a recent common ancestor with *P. axillaris* subsp. *axillaris*. *Petunia toropiensis* Stehmann and Larocca is the most basal species in this group. Such relationships were confirmed in the remaining analyses, especially in the network obtained with SplitsTree, where *P. axillaris* subsp. *axillaris* and *P. secreta* did not differ from each other, and *P. toropiensis* is the most divergent in this group (Supplementary Figure S2).

**Figure 3:**
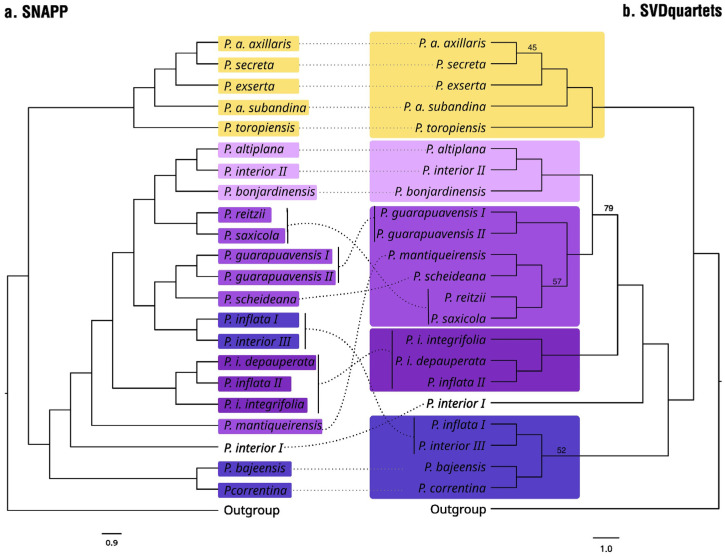
Comparison between SNAPP and SVDQuartets tree topologies. Nodes with bootstrap (BS) < 95% are marked in the SVDQuartets tree. The long corolla tube clade is highlighted in yellow, while short corolla tube lineages are in shades of purple. In the SNAPP tree, corresponding taxa are marked with the same colors. Straight dashed lines indicate congruent topologies, and curved dashed lines highlight incongruences.

The analysis evaluating all possible species quartets (Figure 3b) revealed the same relationships between the species with long corolla tubes, except *P. axillaris* subsp. *axillaris*/*P. secreta* was less supported than in ML (Figure 1). For this clade, the ASTRAL analysis was consistent with the remaining results (Supplementary Figure S3). Just a few gene trees did not follow the species tree in this clade (Supplementary Figure S4).

Regarding the ST, the evolutionary relationships between lineages were more complex, and there were more inconsistencies between methods. In this clade, *P. interior* T. Ando and Hashim. and *P. inflata* R.E. Fr. were not monophyletic, with three and two lineages grouping closer to other species, respectively, in all analyses, and the two lineages of *P. guarapuavensis* T. Ando Hashim grouped only in the ML tree (Figure 1) and network (Supplementary Figure S2). Despite being related, *P. integrifolia* subsp. *depauperata* (R.E. Fr.) Stehmann and *P. integrifolia* subsp. *integrifolia* (Hook.) Schinz and Tell never appeared as sister species, with *P. inflata* II as the sister lineage of *P. integrifolia* subsp. *depauperata*.

The first group to diverge was *P. correntina* Greppi and Stehmann/*P. bajeensis* T. Ando and Hashim. (ca. 0.75 Mya), which was the sister taxon to *P. interior* III/*P. inflata* I in most analyses, despite having low support. *Petunia correntina* occurs only in Corrientes Province in Argentina, while *P. bajeensis* is endemic to Bagé Municipality in southern Brazil. The linear distance between the collection sites of these two species is approximately 800 km. In the ST, several gene trees differed from the species tree (Supplementary Figure S4), and SNAPP and SVDQuartets analyses diverged significantly (Figure 3).

Except for the monophyly of *P. guarapuavensis* (Figure 1) that had low support, accounting for incomplete lineage sorting (Figure S3) and ML-based tree revealed the same relationships between lineages in the ST. In these analyses, *P. mantiqueirensis* T. Ando and Hashim., from the temperate highland grasslands in southeastern Brazil, appeared as the sister of *P. scheideana* L.B. Sm. and Downs, which was the sister of *P. guarapuavensis* in the remaining analyses.

### 2.3. New Taxonomic Circumscription for Petunia Juss.

Considering the current results and previously published suggestions regarding the taxonomic status of the *Petunia* taxa, we propose the following genus composition that morphological traits can identify. The evidence based on morphological and molecular data accumulated in the last two decades, discussed above, supports the acceptance of 20 species instead of the 14 species previously recognized [8]. The increase in species was due to two new taxa recently described, a change in the current taxonomic status of some subspecies, and species resurrected from synonymy. Below, we provide an identification key to the *Petunia* species and a taxonomic synopsis, including synonyms, typification, and changes in the circumscription of some taxa.

### 2.4. Key to the Species

1. Anthers with yellow pollen
**2**
1′. Anthers with lilac or violaceous pollen
**7**
2. Corolla salverform (trumpet-shaped), filaments adnate to the base of the corolla tube 
***P. toropiensis***
2′. Corolla infundibuliform (funnel-shaped), with subcylindrical tube; filaments adnate up to half of the corolla tube
**3**
3. Corolla reddish, anthers and stigma exserted from the corolla tube; sciophilous plants
***P. exserta***
3′. Corolla white, pink, magenta, or purple, anthers and stigma opened at the mouth of the corolla tube; heliophilous plants
**4**
4. Corolla pink, magenta or purple, without perceptible fragrance 
***P. secreta***
4′. Corolla white, with a perceptible fragrance at night 
**5**
5. Corolla tube 30–46 (47) mm long; limb 21–25 mm across 
***P. axillaris***
5′. Corolla tube 45–75 mm long, limb 40–50 mm across 66. Androecium in two lengths, four longer stamens and one shorter (4 + 1) 
***P. parodii***
6′. Androecium in three lengths, two longer, two middle and one shorter (2 + 2 + 1) 
***P. subandina***
7. Corolla with a pink or pink-reddish limb 
**8**
7′. Corolla with a magenta or purple limb 98. Filaments adnate more than 9 mm to the base of the corolla tube; stigma weakly exserted above the anthers of the larger stamens 
***P. saxicola***
8′. Filaments adnate less than 8 mm to the base of the corolla tube; stigma positioned below the anthers of the larger stamens 
***P. reitzii***
9. Stigma positioned at the same height or above the anthers of the larger stamens 
**10**
9′. Stigma positioned below the anthers of the larger stamens 
**13**
10. Corolla campanulate, corolla tube and throat magenta or purple, with violet hue; stigma opening in front of the anthers of the larger stamens 
***P. bonjardinensis***
10′. Corolla infundibuliform or tubular-infundibuliform, corolla tube and throat whitish, with reticulated violet venation; stigma positioned at the same height as the anthers of the larger stamens 
**11**
11. Corolla tube longer than 25 mm, the ratio of the length to the width of the mouth is circa or bigger than 3:1 
***P. mantiqueirensis***
11′. Corolla tube shorter than 20 mm, the ratio of the length to the width of the mouth is circa or smaller than 2:1 
**12**
12. Plant procumbent or ascendent, not climbing; branches and leaves usually pilose; petiole short, up to 5 mm long; fruiting pedicels 2–3 cm long, straight or slightly curved 
***P. guarapuavensis***
12′. Plant with long branches, climbing on the surrounding vegetation, branches and leaves usually glabrous or sparse-pilose; leaves long–petiolate, more than 5 mm long; fruiting pedicels usually more than 4 cm long, markedly curved 
***P. scheideana***
13. Calyx lobed to the middle; plants with prostrate branches, generally growing at ground level, rarely ascending, sometimes rooting at the nodes 
**14**
13′. Calyx deeply lobed; erect plants or with ascending branches, sometimes prostrate, never rooting at the nodes 
**15**
14. Rooting plants, with pilose branches and leaves; membranous leaves; spatulate to orbicular 
***P. altiplana***
14′. Plants generally not rooting, with glabrous or glabrescent branches and leaves; somewhat fleshy leaves, elliptical to narrowly elliptical 
***P. dichotoma***
15. Pedicel inflexed in fruiting 
**16**
15′. Pedicel reflexed in fruiting 
**18**
16. Corolla limb small, 18–20 mm in diameter, base of corolla tube cylindrical, stigma bilobed
***P. occidentalis***
16′. Corolla limb normally greater than 20 mm in diameter, corolla tube funnel-shaped, stigma not bilobed
**17**
17. Corolla limb 30–40 mm in diameter, interior corolla tube pale purple, filaments incurved at the apex
***P. inflata***
17′. Corolla limb 18–29 mm in diameter, interior corolla tube whitish-green, apex of longer filaments nearly straight, apex of medium filaments curved laterally and opposite each other
***P. correntina***
18. Anthers with canaliculate thecae upon dehiscence 
***P. interior***
18′. Anthers with flat thecae, fully opening upon dehiscence
**19**
19. Viscid plants; leaves with prominent venation; opening of corolla tube reniform in frontal view 
***P. bajeensis***
19′. Plants not evidently viscid; leaves with obscure venation; opening of corolla tube elliptical in frontal view
***P. integrifolia***


### 2.5. Taxonomic Synopsis

Herbaria abbreviation follows the Index Herbariorum (https://sweetgum.nybg.org/science/ih/, accessed on 10 March 2025).

1. ***Petunia altiplana*** T. Ando and Hashim., J. Linn. Soc., Bot., 111: 269. Fig. 3–4. 1993. Type: Brazil. Rio Grande do Sul: Cambará do Sul, 9.2 km NE of Tainhas to Cambará do Sul, 940 m, 30.Nov.1991, T. Ando, G. Hashimoto and S. Iida B319 (holotype S #S-04-3116!, isotype US [00386152] image!).

Geographic distribution: Brazil. In the highlands of Santa Catarina and Rio Grande do Sul states.

2. ***Petunia axillaris*** (Lam.) Britton, Sterns and Poggenb., Prel. Cat.: 38. 1888. *Nicotiana axillaris* Lam., Tabl. Encycl. 2: 7. 1793. ≡ *Stimoryne axillaris* (Lam.) Wijsman, Acta Bot. Neerl. 34: 347. 1985. Type: Uruguay. Montevideo, s.d., Commerson s.n. (lectotype P [P00357810]!; designated by Stehmann and Greppi 2013).

*=Petunia nyctaginiflora* Juss., Ann. Mus. Natl. Hist. Nat. 2: 215. Tab. 47, fig. 2. 1803. ≡ *Nicotiana nyctaginiflora* (Juss.) Lehm., Gen. Nicot. Hist.: 47. 1818. ≡ *Nicotiana axillaris* var. *nyctaginiflora* (Juss.) Kuntze, Rev. Gen. Plant. 3 (2): 223. 1898. Type: Uruguay. Montevideo, s.d., Commerson s.n. (lectotype P [P00675648]!, here designated).

=*Petunia propiqua* Miers, London J. Bot. 5: 185. 1846. *Nicotiana axillaris* var. *propinqua* (Miers) Kuntze, Rev. Gen. Plant. 3 (2): 223. 1898. Type: Argentina. Buenos Aires: s.d., J. Miers 730 (holotype BM [000992203]!).

Geographic distribution: Argentina, Brazil, Uruguay. The species is distributed from the eastern Rio Grande do Sul, Brazilian state, to Buenos Aires, La Plata and Rio Negro Provinces, in Argentina. In Uruguay, it can be found in southern departments, between the Rio Negro River and La Plata River.

Nomenclatural note: Stehmann and Greppi [30] cited as the holotype of *P. nyctaginiflora* the specimen housed in the general herbarium (P, [P00475689]) at the Muséum National d’Histoire Naturelle, collected by Commerson in Montevideo, Uruguay. As there are other specimens, collected by Commerson from the same locality in P, LINN, and MPU, it is necessary to choose one of them to serve as the lectotype [31]. Among the Commerson collections, we identified a specimen labeled as part of Jussieu’s herbarium (P, [P00675648]), which corresponds closely to the description and the illustration provided in the protologue, including a dissected corolla depicted in plate XLVII, figure 2a–c. The herbarium sheet is a single gathering with six fragments, all morphologically similar and belonging to the same taxon. This specimen is here designated as the lectotype of *P. nyctaginiflora*, representing unambiguously the original material and being the most appropriate choice. If the specimen from MPU [MPU020156], or part of the gathering, should be considered isolectotype, a deeper historical analysis must be done.

3. ***Petunia bajeensis*** T. Ando and Hashim., Brittonia 50(4): 483. 1998. Fig. 1–2. Type: Brazil. Rio Grande do Sul. Mun. Bajé: Rte. BR153, 7 km S of the south entrance of Bajé to Aceguá, 31°26′26″ S, 54°08′17″ W, 14.Nov.1994, G. Hashimoto, T. Ando and N. Akiba B796 (holotype MBM [MBM240688]!, isotypes BM [BM000583299]!, K [K000585305]!, L [L0538649] image!, MVFA!, R [R000211340]!, S [S04-3117] image!, SI [SI004084]!, SP [SP001633]!, U [U0008242] image!, US #3386855 [00604164] image!).

Geographic distribution: Brazil. The few known populations occur in a small area in the southernmost Rio Grande do Sul state.

4. ***Petunia bonjardinensis*** T. Ando and Hashim., J. Linn. Soc., Bot., 111: 266. Fig. 1–2. 1993. Type: Brazil. Santa Catarina. Bom Jardim da Serra: Route SC56, 1 km W of Bom Jardim da Serra to Mantiqueira, 1240 m, 1. Nov.1990, T. Ando, G. Hashimoto and K. Buto B170 (holotype S #S08-5396!, isotypes BM [BM000992199]!, US [US00386151] image!).

Geographic distribution: Brazil. Endemic to a small area near the border of the Southern Brazilian Plateau, Santa Catarina state.

5. ***Petunia correntina*** Greppi and Stehmann, Phytotaxa 414(6): 290. 2019. Type: Argentina. Corrientes. Dep. Goya. Ruta Nacional 12, km 832, tramo de ruta entre la ciudad de Goya y la Ruta Provincial 24, costado del camino, suelo arenoso. Coordenadas: 29,1759S, 58,8739W, Altitud 56 m. 30.Nov.2017, flor y fruto, J. A. Greppi, J. C. Hagiwara and S. Otomo 1581 (holotype BAB!, isotypes BHCB [BHCB201078]!, ICN [00043982]!, MBM [MBM436640]!, RB [RB01443739]!).

Geographic distribution: Argentina. It is only known from a few localities in the Corrientes Province.

6. ***Petunia dichotoma*** Sendtn., Fl. Bras. 10: 173. 1846. Type: Brazil. In Brasilia Australi. Sellow s.n. (lectotype P[P00724302] image!; here designated).

=*Petunia violacea* subsp. *depauperata* R. E. Fr., Kongl. Svenska Vetenskapsakad. Handl. 46 (5): 34. 1911. ≡ *Petunia integrifolia* var. *depauperata* (R. E. Fr.) L. B. Sm. and Downs, Fl. Ilustr. Cat. (Solan.): 266. 1966. ≡ *Petunia integrifolia* subsp. *depauperata* (R. E. Fr.) Stehmann, Fl. Ilustr. Cat. (Solan.): 266. 1966. Type: Brazil, Rio Grande do Sul, Vieira prope Rio Grande oppidum, in campis collibusque arenae mobilis, 25.Nov.1892, Lindman 831 (lectotype S!; designated by Stehmann and Bohs [16].

=*Petunia littoralis* L. B. Sm. and Downs, Fl. Ilustr. Cat. (Solan.): 269. Fig. 27a,h–i. 1966, syn. nov. ≡ *Stimoryne littoralis* (L. B. Sm. and Downs) Wijsman, Acta. Bot. Neerl. 34 (3): 348. 1985. Type: Brazil. Santa Catarina. Florianópolis: Rio Vermelho, restinga, 2 m, 6.Oct.1964 (fl,fr), Klein, Souza Sob. and Bresolin 5852 (holotype US [US00067624]!, isotypes BHCB [BHCB035247]!, HBR #31857!, FLOR [FLOR0001097]!). Syn. nov.

Geographic distribution: Brazil. It spans the coastal plain from Santa Catarina Island in Santa Catarina state to the southernmost Rio Grande do Sul state.

Nomenclatural note: The original material of *P. dichotoma* examined by Sendtner in the Herbarium Berolinense (B) was destroyed, leaving only a photograph [=image F neg. 39272!]. A duplicate kept in herbarium P [P00724302] was designated as the lectotype.

The original material collected by Sellow and deposited in herbarium B [=F neg. #39272 image!], used in the description of *P. dichotoma* [32] was destroyed. A duplicate of this material [P00724302] was found in the herbarium of the Muséum National d’Histoire Naturelle and was chosen as the lectotype.

Two typifications were proposed based on illustrations linked to the protolog. For *Petunia integrifolia,* we chose the illustration (Table 3113) of the basionym *Salpiglossis integrifolia* Hooker [33] in the protologue, that represents well the species. The original material examined was based on a cultivated plant at Glasgow Botanic Garden, obtained from seeds brought from Buenos Aires by John Tweedie in the autumn of 1830. Three years later [34], this taxon was described and illustrated as *P. violacea* based on plants obtained from Buenos Aires, and employed as the valid name of the species in horticultural and genetic literature for decades [8].

7. ***Petunia exserta*** Stehmann, Napaea 2: 19. 1987. Type: Brazil. Rio Grande do Sul. Caçapava do Sul: Guaritas (fl,fr), M. Sobral 4290 (holotype ICN #134201 [00000619]!, isotypes B [B_10_0248776] image!, BHCB #162272 [BHCB5531]!, L [L0003612] image!, NY [1795760] image!, SP [SP001634]!, RB [RB00719749]!).

Geographic distribution: Brazil. The distribution is limited to the south-central region of Rio Grande do Sul state.

8. ***Petunia guarapuavensis*** T. Ando and Hashim., Brittonia 46 (4): 340. Fig. 1–2. 1994. Type: Brazil. Paraná. Guarapuava: Rt. BR373, 30 km E of Guarapuava to Relógio, 1260 m, 6.Dec.1989, G. Hashimoto, T. Ando and H. Watanabe B65 (holotype MBM [MBM182740]!, isotypes BM [000992205]!, HBR (not seen), S #S08-5394 image!, US [00516695] image!).

Geographic distribution: Argentina, Brazil. It occurs in the Misiones province in Argentina and the inland regions of Paraná and Santa Catarina states in southern Brazil.

9. ***Petunia inflata*** R. E. Fr., Kongl. Svenska Vetenskapsakad. Handl. 46 (5): 35. Tab. 2, f. 1; tab. 5, f. 4a–c. 1911. ≡ *Stimoryne integrifolia* (Hook.) Wijsman subsp. *inflata* (R. E. Fr.) Wijsman, Acta. Bot. Neerl 34 (3): 347. 1985. ≡ *Petunia integrifolia* (Hook.) Schinz and Thell. subsp. *inflata* (R. E. Fr.) Wijsman, Acta Bot. Neerl. 31 (5–6): 484, 1982. Type: Paraguay. Tobaty: in dumetis collium, Set.1900, Hassler 6146 (lectotype K [K000585308]!, isolectotype W [1904-0000792]!; designated by Stehmann and Greppi [30]).

Geographic distribution: Argentina, Brazil, Paraguay. It ranges from southern Paraguay and Misiones Province in Argentina to northern and northwestern Rio Grande do Sul in Brazil.

10. ***Petunia integrifolia*** (Hook.) Schinz and Tell., Vierteljahrsschr. Naturf. Ges. Zürich 60: 361. 1915. ≡ *Salpiglossis integrifolia* Hook., Bot. Mag. 58. Tab. 3113. 1831. *Nicotiana integrifolia* (Hook.) O. Kuntze, Rev. Gen. Pl. 3 (2): 223. 1898. ≡ *Stimoryne integrifolia* (Hook.) Wijsman, Acta. Bot. Neerl. 34 (3): 347. 1985. Type: Cultivated in Glasgow, seeds from Buenos Aires, Argentina [illustration] Hooker, Bot. Mag. 58. Tab. 3113 (lectotype, here designated).

=*Nierembergia phoenicea* G. Don in Sweet, Brit. Fl. Gard. 2: 193. 1833. Type: Not located.

=*Petunia violacea* Lindley, Edwards’s Bot. Reg. 19: Tab. 1626. 1833. Type: A native from Buenos Aires, Argentina (specimen not located). [Illustration] Lindley, Edwards’s Bot. Reg. 19: Tab. 1626. (lectotype, here designated).

=*Stimoryne purpurea* Raf., Fl. Tell. 3: 76. 1836. Type: Not located.

=*Petunia riograndensis* T. Ando and Hashim., Brittonia 50: 485 (Fig. 3–5). 1998. Type: Brazil. Rio Grande do Sul: Mun. São Jerônimo: 39 km SSW from Morrinhos to Palmeira, 30°27′01″ S, 51°58′01″ W, 25.Nov.1994, G. Hashimoto, T. Ando and N. Akiba B860 (holotype MBM [MBM240702]!, isotype S #S04-3126 image!).

Geographic distribution: Argentina, Brazil, and Uruguay. In Argentina, it is found in the southeast part of the Mesopotamian region, primarily in areas near the Uruguay River. In Brazil, the distribution is mainly located in the southern half of Rio Grande do Sul.

Nomenclatural notes: Two lectotypifications performed here designate illustrations as lectotypes. *Salpiglossis integrifolia* Hooker, the basionym of *Petunia integrifolia*, was described based on plants grown in Glasgow, and no specimens could be found in herbaria. For *Petunia violacea* Lindley, also described based on cultivated material, there is a lack of material in herbaria. Since there is a good illustration in the protologue of both taxa, which matches the morphological description presented, we chose them as lectotypes, following Article 9.3 of the *International Code of Nomenclature for algae, fungi, and plants* [31].

11. ***Petunia interior*** T. Ando and Hashim., Brittonia 48 (2): 217. Fig. 1–4. 1996. Type: Brazil. Santa Catarina: Mun. Chapecó, 15 km SW from Chapecó to Nonoai (Rio Grande do Sul), 27°13′26″ S, 52°40′08″ W, 26.Nov.1993, Hashimoto, Ando, Tanaka and Tsukamoto B569 (holotype MBM [MBM199111]!, isotypes BM [BM000992202]!, R [R000211356]!, S #S-R-7669 image!, US [US00512894] image!).

Geographic distribution: Argentina, Brazil. It ranges from Misiones province in Argentina to the northwestern Rio Grande do Sul and western Santa Catarina state in southern Brazil.

12. ***Petunia mantiqueirensis*** T. Ando and Hashim., Brittonia 46 (4): 340. Fig. 1–2. 1994. Type: Brazil. Minas Gerais: Camanducaia, 22 km SE from Camanducaia to Monte Verde, 22°48′29″ S, 46°04′46″ W, 1300 m, 7.Dec.1991, G. Hashimoto, T. Ando and S. Iida B357 (Holotype S #S-R-4407!; isotypes BM [BM000992204]!, K [K000585313]!, S #S08-5398 image!, SP [SP001636]!, U [U0006792 ] image!, US [US00433341] image!).

Geographic distribution: Brazil. It is endemic to the southern part of Minas Gerais state, in southeastern Brazil.

13. ***Petunia occidentalis*** R. E. Fr., Kongl. Svenska Vetenskapsakad. Handl. 46 (5): 37–38. Tab.2, f.5; tab. 5, f. 5a–c. ≡ *Petunia integrifolia* subsp. *occidentalis* (R. E. Fr.) Wijsman, Acta Bot. Neerl. 31 (5–6): 484. 1982. ≡ *Stimoryne integrifolia* subsp. *occidentalis* (R. E. Fr.) Wijsman, Acta. Bot. Neerl. 34 (3): 347. 1985. Type: Bolívia. Bermejo: 18.nov.1903, K. Fiebrig 2135 (lectotype MO #172173 not seen; isolectotypes BM!, K!; designated by Stehmann and Greppi [30]).

Geographic distribution: Argentina and Bolivia. It is confined to the Sub-Andean region in northwestern Argentina and southern Bolivia.

14. ***Petunia parodii*** Steere, Pap. Michigan Acad. Sci. 13: 213. Pl. 32–34. 1931. ≡ *Stimoryne axillaris* subsp. *parodii* (Steere) Wijsman, Acta Bot. Neerl. 34 (3): 347. 1985. Type: Argentina. Formosa. Cultivated from seeds collected by L. R. Parodi, in the central part of the province of Formosa in the north of Argentina, Steere 202-1 (holotype MICH #1109912 image!).

Geographic distribution: Argentina, Brazil, Paraguay, and Uruguay. It is widely distributed from southern Paraguay and northeastern Argentina to northern Uruguay and the western part of the Rio Grande do Sul state in southern Brazil.

15. ***Petunia reitzii*** L. B. Sm. and Downs, Phytologia 10: 439. Tab. 11, fig. 5–6. 1964. *Stimoryne reitzii* (L. B. Sm. and Downs) Wijsman, Acta. Bot. Neerl: 34 (3): 347. 1985. Type: Brazil: Santa Catarina. Bom Retiro: Riozinho, 1000 m, 24.Dec.1948, R. Reitz 2760 (holotype US [US00067630]!, isotype HBR #5225!).

Geographic distribution: Brazil. It is confined to a small region along the eastern border of the southern Brazilian plateau, specifically in Santa Catarina state.

16. ***Petunia saxicola*** L. B. Sm. and Downs, Phytologia 10: 439. Tab. 11, fig. 7–8. 1964. ≡ *Stimoryne saxicola* (L. B. Sm. and Downs) Wijsman, Acta. Bot. Neerl. 34 (3): 347. 1985. Type: Brazil. Santa Catarina. Lages: On Rock, Alto da Serra, Encruzilhada, alt. 900 m, 30.Oct.1962 (fl), R. Reitz and R. M. Klein 13931 (holotype US [US00067629]!, isotypes BHCB [BHCB035258]!, HBR #52485!, R!).

Geographic distribution: Brazil. It is only known from a small area on the eastern border of the southern Brazilian plateau, in Santa Catarina state.

17. ***Petunia scheideana*** L. B. Sm. and Downs, Phytologia 10: 439. Tab. 11, fig. 9–10. 1964. *Stimoryne scheideana* (L. B. Sm. and Downs) Wijsman, Acta. Bot. Neerl. 34 (3): 348. 1985. Type: Brazil. Santa Catarina. Campo Alegre: Fazenda superior de Ernesto Scheide, 900–1100 m, 9.Nov.1956, L. B. Smith and R. M. Klein 7522 (holotype US [00067628] image!, isotypes BHCB [BHCB035260]!, HBR #30943!, R [R000130006]!, NY [00138813] image!, R [R000130006]!).

Geographic distribution: Brazil.

18. ***Petunia secreta*** Stehmann and Semir. Monogr. Syst. Bot. Missouri Bot. Gard. 104: 346 (–348; fig. 3). Type: Brazil. Rio Grande do Sul. Caçapava do Sul: Pedra do Segredo, 2.Nov.1995 (fl,fr), J. R. Stehmann 2101, J. Semir and J. Dutilh (holotype UEC #77965!, isotypes BHCB!, MBM!).

Geographic distribution: Brazil. It is restricted to the south-central part of the Rio Grande do Sul state.

19. ***Petunia subandina*** (T.Ando) Stehmann and Freitas, ***comb. and stat. nov*.** *Petunia axillaris* subsp. *subandina* T. Ando, Acta Phytotaxonomica et Geobotanica, 7(1): 21, 1996. Type: Argentina. Pro. Jujuy, Dept. Dr. M. Belgrano, Route 9, Leon, 24°1′4″ S, 65°26′39″ W, 13.Nov.1991, S.Iida, T.Ando A100 (holotype SI [SI004077]!, isotype S #S-R-7677 image!).

Geographic distribution: Argentina, Bolivia. It is found in the sub-Andean region, as well as in La Pampa, San Luis, Mendoza, Salta, and Jujuy provinces of Argentina, along with southern and central Bolivia.

20. ***Petunia toropiensis*** Stehmann and Larocca, *Acta Bot. Brasil*., 37-e20220266: 2 2023. Type: Brazil. Rio Grande do Sul: São Martinho da Serra, estrada para a antena, 29°27′8.44″ S, 54°05′21.96″ W, 197 m, 9.Nov.2021 (fl.,fr.), J.R. Stehmann, J. Larocca and R. Vasconcelos 6557 (holotype BHCB [BHCB206043]!, isotypes ICN!, MBM!).

Geographic distribution: Brazil. It is only known for a few sites in the central region of the Rio Grande do Sul state.

## 3. Discussion

We analyzed the currently accepted *Petunia* taxa (POWO; https://powo.science.kew.org/, accessed on 7 May 2025) using high-throughput genomic coverage and evaluated the morphological variation to revisit the infrageneric taxonomy and the evolutionary relationships among the lineages of this genus. We also considered previously published works detailing genetic polymorphisms and ecological relationships between these taxa.

Phylogenomic studies have provided better resolution at and below the species level [35,36]. Among polytypic species, the number of subspecies typically fluctuates over time and among authors, particularly because of the difficulties in identifying taxonomic boundaries between recently divergent groups, which often maintain incomplete reproductive isolation [37]. Phylogenomics can distinguish between closely related organisms and help in resolving both short and long tree branches [38].

Many relationships observed through DArT-seq align with prior studies based on limited genomic coverage [11] and other data types [39,40], while others undergo further review. All analyses categorize the genus into two clades, fully supported by corolla tube length. This categorization was previously suggested, despite the inconsistent positioning of *P. occidentalis* in earlier research [11]. Besides tube length, species differ in pollen color; those in the LT, excluding *P. occidentalis*, show yellow pollen, while species with short corollas exhibit blue pollen [8]. The two clades diverge through evolutionary processes, shaped by different selective forces and geographic distributions [23].

Like phylogenetic trees based on partial genome coverage, our results revealed some incongruences, particularly when comparing different analysis methods (Figure 3). Although these incongruences were less pronounced than in previous studies, they support the hypothesis of incomplete lineage sorting (ILS) and, in some instances, suggest hybridization.

Considering the evolutionary relationships between clades and species (Figure 1), the timing of lineage divergence (Figure 2), and the geographic distribution of taxa [8], our data revealed the crucial role of migration in species diversification. All divergence times coincide with the Pleistocene period (Figure 2), which significantly influenced the landscape design of southern South American grasslands [41], characterized by alternating periods of contraction and expansion of species’ ranges. Colder and drier cycles favored the expansion of species adapted to the grasslands, which became fragmented and contracted their distributions as forests advanced into the open fields during warmer and wetter periods. This successive alternation led to the isolation of lineages, facilitating the observation of ILS [40] and resulting in differentiation in allopatry [15]. Moreover, the increasing distribution potentially promoted secondary contacts between partially or fully differentiated lineages that, along with the weak barriers to gene flow between *Petunia* species [42], could facilitate hybridization [19,27]. More than those from the lowlands, the highland species show lineage divergence in allopatry as *P. reitzii* L.B. Sm. and Downs/*P. saxicola* L.B. Sm. and Downs or *P. altiplana* T. Ando and Hashim-*P. interior* II/*P. bonjardinensis* T. Ando and Hashim (Figure 1). Despite high polymorphism sharing, these species differ in distribution and occupy various microenvironments [8], including a wide altitudinal range.

The main clades’ delimitation coincides with striking morphological features, the corolla tube length and pollen color. The only exception is the *P. occidentalis*, for which we have lost information. Previous studies included *P. occidentalis* and relied solely on plastid markers or a few plastid and nuclear fragments, which placed this species in the LT despite its short corolla tube and blue pollen. This rare species has a limited distribution, inhabiting the sub-Andean region in Argentina, which may have mistakenly positioned it as the sister group of *P. axillaris* subsp. *subandina* due to the strong biogeographical signal of these genetic markers in *Petunia* [11,12,43]. The status of this species is pending further analysis.

The remaining species of the LT have evolved under the strong influence of hybridization [22,26,28,44] and local adaptation [22,45]. These species primarily diverge in morphology following their floral syndromes [23]. The tree based on DArT-derived markers reproduced the evolutionary relationships previously observed for this clade, despite lacking information on *P. axillaris* subsp. *parodii* and *P. occidentalis*. The positioning of these lineages agrees with previously proposed hypotheses [25,43] for their diversification and reinforces other findings, such as the differentiation between *P. secreta* and *P. axillaris* subsp. *axillaris* being recent [43] and attributed to only the flower color [46] due to the regain of a gene function [47] which promotes the transition from hawkmoth to bee pollination in *P. secreta* [48].

The divergence of *P. exserta* from the other species could be related to the ecological features of this species, as *P. exserta* inhabits a unique environment, inside shaded caves on shallow soil completely inhospitable for other *Petunia* species [49]. Some analyses [11,40,43] positioned *P. exserta* externally in this clade, whereas others reveal the close relationships between *P. exserta* and *P. axillaris* subsp. *parodii* [39,50] (where this subspecies was named only as *P. axillaris*) or the exclusive polymorphisms shared between them [51].

The subspecies of *P. axillaris* do not share a recent common ancestor, suggesting that all should be elevated to species status. Multilocus data analysis using complex methods revealed the diversity of multiple species in *Petunia axillaris*. Phylogenetic analysis showed that *P. axillaris* is polyphyletic, composed of three evolutionary lineages [43]. Haplotype distribution and genetic differentiation analysis detected strong structure and high levels of genetic differentiation among subspecies [17]. Geographic distance did not explain the genetic differentiation related to morphological and ecological divergence [17,52]. The coalescent multispecies analysis supports the recognition of three lineages with sufficient morphological characters to elevate their status to the species level. Here, we show that the individuals named *P. axillaris* subsp. *axillaris* differ from those classified as *P. axillaris* subsp. *subandina* (Figure 1). Thus, the three white-flowered lineages are independent species: *P. axillaris*, *P. parodii*, and *P. subandina*.

We accept a restricted circumscription to *P. axillaris*, including the populations with tubular white flowers, fragrant in the evening, with corolla shorter than that of the two morphologically related species, *P. parodii* and *P. subandina.* The androecium with anthers positioned in three levels is a trait shared with *P. subandina*. The *P. axillaris* geographic distribution is associated with the Pampean region, in southern and southeastern Rio Grande do Sul (Brazil), Uruguay, and Argentina. The type material of this taxon comes from Montevideo, Uruguay, from where seeds were taken and cultivated in Europe in the 19th century. The crossing of this species with *P. integrifolia* gave rise to the first known hybrids cultivated as ornamental plants (*P.* × *atkinsiana*).

*Petunia parodii* features a long tubular corolla measuring 45–75 mm in length, with distinct stamen lengths of four sub-equal and one shorter (4 + 1). Its geographic distribution ranges from Paraguay and central and northeastern Argentina to southern Brazil and Uruguay, where it inhabits grasslands and disturbed areas, such as roadsides, primarily occupying the lowlands within the Chaco and Pampa ecosystems.

*Petunia subandina* has the largest corolla in the genus, measuring over 10 cm in length. It is related to *P. axillaris and* is found in Bolivia and the foothills of Central and Northeastern Argentina.

The present study was the first molecular analysis to include *P. toropiensis*, a micro-endemic species from the central region in Rio Grande do Sul, a Brazilian state [10]. With floral morphology suggestive of a melitophilous pollination syndrome, this species was shown to be basal to the remaining species and the first lineage to diverge in the clade (Figure 2). This finding reinforces the proposition that LT emerged in the Pampa [42] and, as its distribution expanded [25], it diversified through local adaptation [22,23] or even as a result of transitions in the pollination syndromes [47,48,53].

In the ST, the subspecies of *P. integrifolia* were not sister species, similar to *P. axillaris*. These taxa were compared based on classical phylogenetic markers and under-population diversity markers. Despite being proximately related, these taxa diverged more from each other than from other distantly related taxa [20,21]. These taxa differ in their evolutionary patterns [18,24,54], independently of the genetic marker employed. Morphological and ecological features support elevating them to species status. We proposed to consider *P. integrifolia* subsp. *depauperata* as a valid species named *Petunia dichotoma* Sendtn. This lineage differs from *P. integrifolia* based on the morphologically distinct growing pattern, with long and prostrate branches, glabrous stems and leaves, or almost so, leaves fleshy, narrow, and flowers with calyx middle lobed, inhabiting sand soils throughout the Quaternary sand soils of Santa Catarina and Rio Grande do Sul Brazilian states. In turn, *P. integrifolia* shows shorter decumbent or ascendent branches, pilose stems and leaves, membranaceous leaves, usually elliptic or large-elliptic, and flowers with calyx deeply lobed, inhabiting rocky soils or derived of them, in the mainland of the Pampean region, in the Rio Grande do Sul Brazilian state, Argentina, and Uruguay.

Phylogeographic studies with populations of *P. dichotoma* (still treated as *P. integrifolia* subsp. *depauperata*) throughout its distribution [18,24,54] showed the existence of three major genetic lineages whose geographical distribution was related to different transgression or regression events that occurred in the South Atlantic Coastal Plain during the Pleistocene. These lineages were independent of *P. integrifolia* and correspond taxonomically to *P. dichotoma*, a group accepted here as a distinct species.

The coastal plain populations from Rio Grande do Sul and Santa Catarina were previously recognized as a distinct geographic subspecies of *P. integrifolia* and treated as *P. violacea* Lindl. [55]. The northernmost coastal populations of the species, growing in the sandbanks of Florianópolis Island, in Santa Catarina (Brazil), were described as a distinct species, named *P. littoralis* L.B. Sm. and Downs, and are here accepted as synonyms of *P. dichotoma*.

Initially recognized as independent species [12], *P. guarapuavensis* and *P. scheideana* were synonymized and treated as *P. scheideana* [8], primarily due to their vegetative and floral similarity, particularly in the shape and reticulation of the corolla and the position of the anthers and stigma at anthesis, which are positioned at the same height [8,9]. Plastid polymorphisms revealed two distinct lineages, despite sharing ancestral polymorphism [15]. The phylogenetic tree, constructed using low genomic coverage along with plastid and nuclear markers obtained through Sanger sequencing [11], indicated the independence of these species. A population genetic analysis based on nuclear microsatellites and plastid haplotypes [56] reinforced the separation between these taxa. A study employing DArT-derived markers for multiple populations throughout the entire distribution of *P. guarapuavensis* and *P. scheideana* [19] suggested that there are two independent species. Moreover, this work revealed that the morphological cline leading to the merging of the taxa is due to natural hybridization.

So, based on genetic and ecological evidence, we have decided to resurrect *P. guarapuavensis*, even though the two species are not easily distinguished in the herbarium, suggesting a need for a detailed morphological study of the group, particularly by including more morphological traits such as leaf morphology [3] or floral geometric morphometric evaluation [29,57], which have been successful in identifying closely related species of Solanaceae. The distinct geographic distributions further support recognizing two separate taxa. While *P. guarapuavensis* is found more inland, occurring in the Guarapuava High Plateau in the Paraná state of Brazil and adjacent areas in Santa Catarina, extending to the province of Misiones in Argentina, populations of *P. scheideana* are restricted to the highlands in the Serra Geral in Santa Catarina, closer to the Atlantic coast.

The remaining species in ST have no taxonomic issues, and their evolutionary relationships mostly reflect the altitudinal gradient where they occur, independently of their geographic distribution. For instance (Figure 1), *P. correntina* and *P. bajeensis*, two micro-endemic species that occur in elevations close to sea level, are the first group to diverge in this clade (Figure 2) and are distant ca. 800 km from each other. *Petunia reitzii* and *P. saxicola*, other narrowly distributed species that inhabit the same rock formation in southern Brazil, diverged ca. 0.2 Mya, have high levels of polymorphism sharing [58], but occupy different microenvironments [8]. These species pair exemplify the marked ILS in the genus [40] and the center-peripheral diversification process [25].

The phylogenetic trees obtained from DArT-seq data clarified the relationships within *Petunia* and strongly supported taxonomic decisions that had previously only been suggested. Furthermore, these analyses raised several questions that require further investigation, such as the non-monophyly of *P. interior* and *P. inflata*, for which multiple lineages with different geographic origins and no morphological divergence were observed. The two lineages observed in *P. guarapuavensis* (Figure 3) can be attributed to a phylogeographic barrier as a river crosses their distribution splitting them [19].

Despite our results reinforcing the importance of basing evolutionary analyses and taxonomic decisions on large genomic coverage and multiple individuals per taxon [59], further studies should be conducted to improve the *Petunia* evolutionary tree, solve some open questions on multiple lineages per species, and include the two taxa we lost here. Additionally, some population diversity and structure have highlighted intraspecific variation in other taxa [19,60], which were not contemplated here, and suggest a more complex system. Including more individuals per lineage and all intraspecific lineages per species could help to clarify the species boundaries [61].

## 4. Materials and Methods

### 4.1. Samples

We sampled healthy leaves from at least two individuals of each *Petunia* taxon, preferably from the type location or as close as possible. This ensured that all sampled individuals accurately represented the species’ morphology according to the original description. Additionally, we included more than two individuals for taxa that exhibited multiple evolutionary lineages in previous studies (see Supplementary Table S2 for more details). We dehydrated the leaves in silica gel and powdered them with liquid nitrogen to extract DNA using the CTAB method (Sigma-Aldrich Chem. Co., Ltd., St. Louis, MO, USA) [62]. We estimated DNA concentration and quality using a Qubit Fluorometer (Thermo Fisher Scientific Co., Ltd., Waltham, MA, USA) and a NanoDrop DN-1000 Spectrophotometer (Thermo Fisher), respectively. We considered samples with 260/280 and 260/230 ratios greater than 1.8 as high quality and diluted all to equivalent concentrations.

We prepared DNA libraries with DArTseq^TM^ [63,64] using a combination of PstI-MseI (New England BioLabs Inc., Ipswich, MA, USA) enzymes [63]. We performed sequencing bulking equimolar sample amounts on the Illumina HiSeq 2500 platform (Illumina Inc., San Diego, CA, USA).

### 4.2. Bioinformatics

We inspected the raw data with FastQC 0.11.7 [65] and Multiqc [66] software and removed barcodes, adapters, low-quality regions (<Q30), and short reads (<50 bases) with FastQ-MCF 1.04.807 [67]. We mapped sequences against the *Petunia* reference genome [7] using BWA 0.7.10-r789 [68] with default parameters. All unmapped reads were removed, and we exported the individual SAM files to a BAM file using the *bamaddrg* utility (https://github.com/ekg/bamaddrg, accessed on 10 March 2025) in SamTools 1.3.1 [69].

We used FreeBayes 1.3.6 [70] to call variants using mapping quality > 30, base quality > 30, and read depth > 10. We filtered and retained only biallelic SNPs with up to 10% missing data and a minimum allele frequency (--maf) of 0.04 using VCFTools 0.1.12 [71]. We removed loci under linkage disequilibrium, setting a minimum site distance of 100 bp (--thin) and keeping only one SNP per read.

### 4.3. Phylogenetic Relationships

Using various phylogenetic approaches, we examined the evolutionary relationships among *Petunia* taxa, with four *Calibrachoa species* (*C. spathulata*, *C. caesia*, *C. humilis*, and *C. ovalifolia*) serving as the outgroup. First, we established a relationship network using the *NeighborNet* method in SplitsTree 4.16 [72], which included all individuals.

We then inferred phylogenetic relationships using *SVDquartets* [73] implemented in PAUP* v.4a [74], evaluating all possible quartets with 1000 bootstrap replicates to generate a bootstrap 50% majority-rule consensus tree.

We also ran *SNAPP* [75], a method based on the multispecies coalescent model for SNP data, to estimate the divergence time between lineages and root trees at the outgroup. For calibration, we constrained the crown divergence between *Petunia* and *Calibrachoa* using a dated phylogeny [76] that establishes the age of this node is 8.49 Ma (95% HPD: 5.5–11.67 Ma). We input as a *log*-normal distribution centered at 8.49 Ma with a standard deviation of 0.14, calculated in BEAUTi, part of the Beast 2.7.7 package [77]. We prepared the input data using the *snapp_prep.rb* script [78], limiting the dataset to 1000 randomly selected SNPs and setting the MCMC chain length to 100,000 iterations. Two independent analyses were performed, and *log*-files and trees were combined using LogCombiner 2.7.7 in Beast. We assessed convergence (effective sample size ESS > 200) using Tracer 1.6 [79] and visualized tree topologies and node heights with DensiTree [80] and FigTree 1.4.4 (https://github.com/rambaut/figtree/, accessed on 10 March 2025).

To obtain maximum likelihood (ML) phylogenetic inferences, we produced a species tree using RAxML-NG 1.0.0 [81] under the GTR + ASC substitution model [82] for SNPs, with 1600 bootstrap replicates. The VCF file containing the SNP matrix was converted to Phylip format using PGDSpider 2.1.1.2 [83], and these ML trees were employed to conduct MSC analyses in ASTRAL 5.7.8 [84].

Incongruences among the trees were quantified using the generalized Robinson-Foulds metric from the TreeDist R package, where zero signifies complete concordance and one indicates total discordance [85].

### 4.4. Taxonomic Treatment

The taxonomic key and species synopsis were based on taxonomic literature [8,30], including the protologues of the recently described species [9,10]. We also examined materials from numerous herbaria visited over the last two decades in Argentina, Brazil, Uruguay, and Europe. In addition to the examined material, images of types (cited as [image!]) were viewed on the Global Plants website (https://plants.jstor.org, accessed on 7 May 2025) and other virtual herbaria. The barcodes associated with the specimens were indicated in square brackets. When necessary, the herbarium curator was contacted.

## Data Availability

All data are included in the main text or available online in the Supplementary Materials. Sequences were deposited in GenBank (https://www.ncbi.nlm.nih.gov/genbank/, accessed on 10 March 2025), and the accession codes are available in Supplementary Table S2.

## Supplementary Materials

The following supporting information can be downloaded at: https://www.mdpi.com/article/10.3390/plants14101478/s1, Figure S1: Incongruences among trees; Figure S2: SplitsTree network; Figure S3: ASTRAL tree; Figure S4: *SNAPP* density tree; Table S1: Sequence information and quality; Table S2: Sampling information.

## Abbreviations

The following abbreviations are used in this manuscript:

DArT	Diversity Arrays Technology
SNP	Single nucleotide polymorphism
ST	Short corolla tube clade
LT	Long corolla tube clade
ILS	Incomplete lineage sorting
ML	Maximum likelihood analysis
CTAB	cetyl-trimethyl ammonium bromide
bp	Base pair
