# Supplementary material for: The Genus Petunia (Solanaceae): Evolutionary Synthesis and Taxonomic Review
Source: Plants (Basel). 2025 May 15;14(10):1478. doi: 10.3390/plants14101478 (PMC12115208; doi:10.3390/plants14101478)
Supplement: Supplementary file 1 [file plants-14-01478-s001.zip › plants-3554073-supplementary.pdf]

The Genus *Petunia* (Solanaceae): Evolutionary Synthesis and Taxonomic Review

Luana S. Soares [1], João R. Stehmann [2,*] and Loreta B. Freitas [1,*]

[1] Department of Genetics, Universidade Federal do Rio Grande do Sul, Porto Alegre 90509-900, Brazil
[2] Department of Botany, Universidade Federal de Minas Gerais, Belo Horizonte 31270-901, Brazil
* Correspondence: stehmann@ufmg.br (J.R.S.); loreta.freitas@ufrgs.br (L.B.F.)

Supplementary Materials

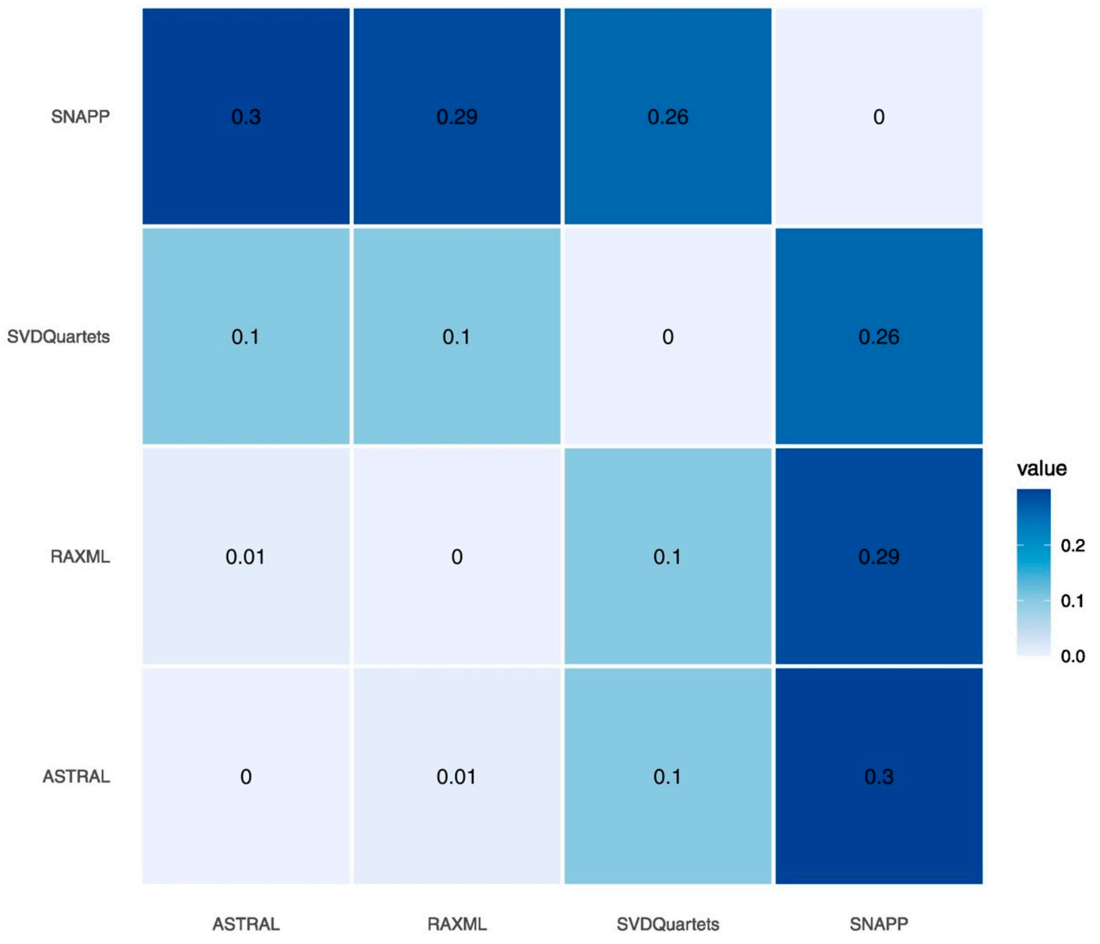

Figure S1. Incongruences among the trees obtained with different methods. Incongruences were quantified using the generalized Robinson-Foulds metric with TREEDIST. Zero signifies complete concordance and one indicates total discordance between methods.

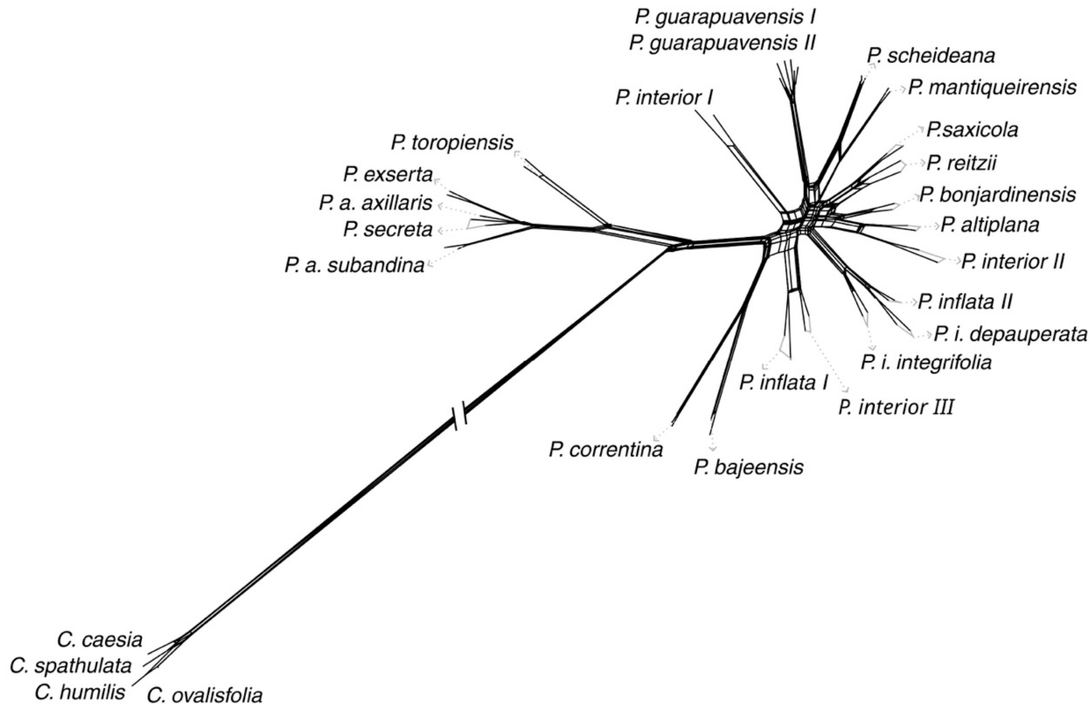

Figure S2. SPLITSTREE network of *Petunia* individuals using the neighbor-joining method. The outgroup (*C. caesia, C. spathulata, C. humilis,* and *C. ovalifolia*) has a trimmed branch for better visualization. At least two individuals per lineage were included. Gray dashed arrows indicate lineage branch placements.

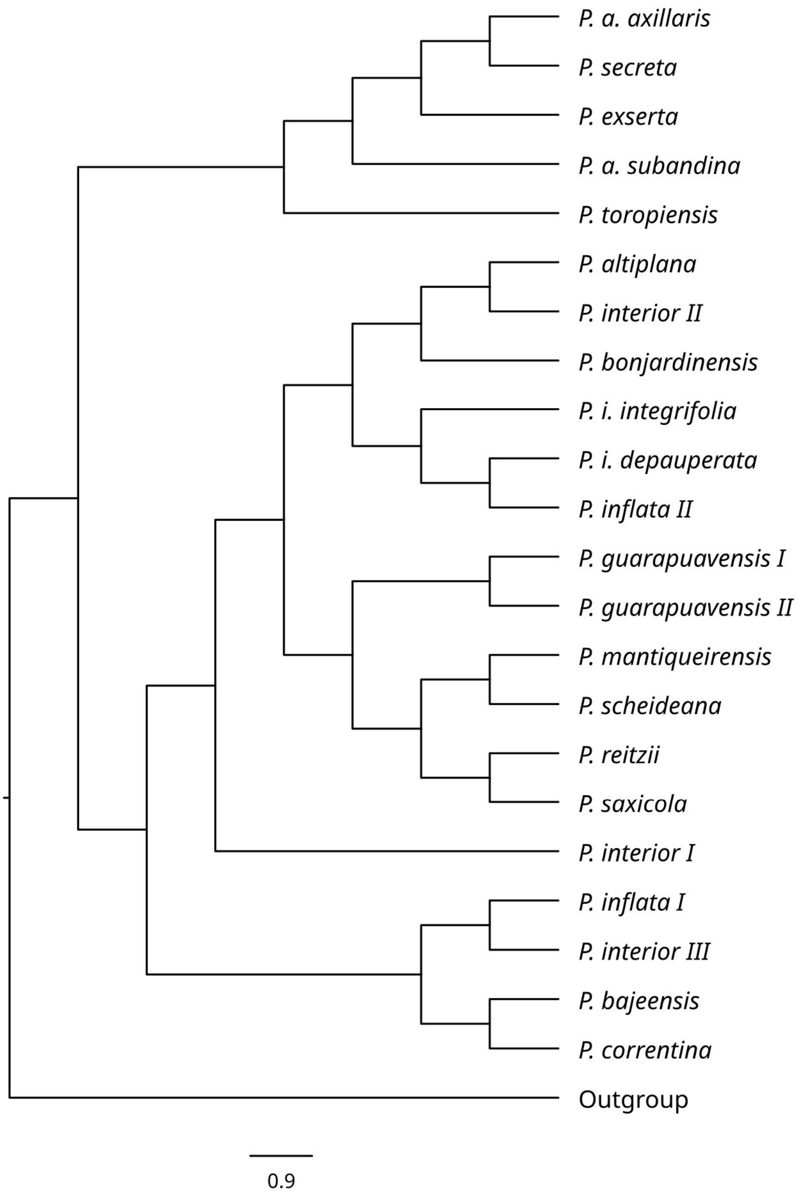

Figure S3. ASTRAL tree of *Petunia* lineages inferred from RAxML gene trees.

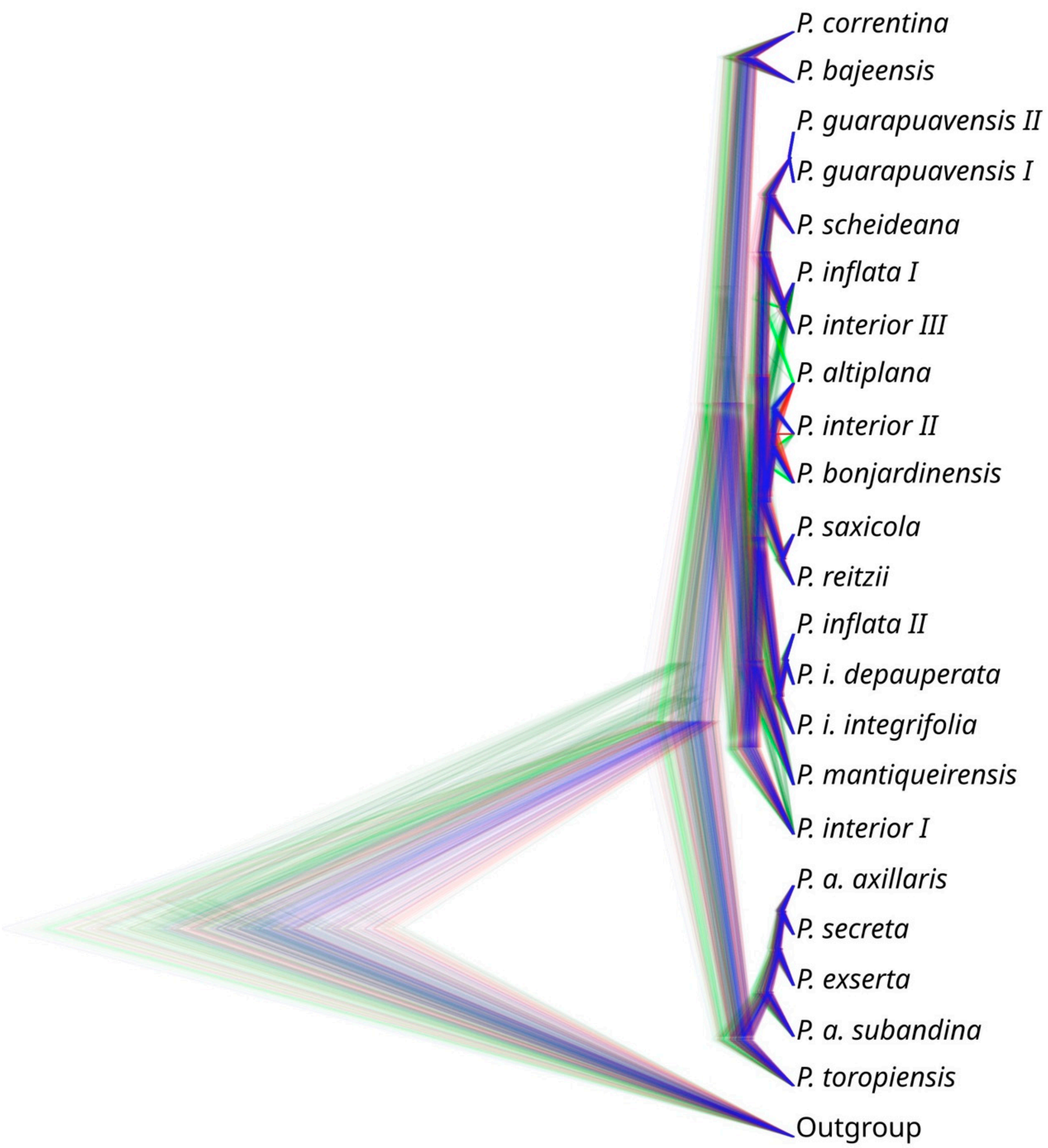

Figure S4. SNAPP density tree illustrating phylogenetic relationships among *Petunia* species. Variability in tree topologies reflects multiple phylogenetic histories.

Table S1. Sequence information and quality

Sequence ID	Barcode	Species	Read pd	Total reads	Filtered	Mapped	%mapping	SNPs	Filtered SNPs	Mean Depth	%Missing data
3006023	GACGTGAGGCTCTAT	*C. spatulatha*	138	565,167	419,628	299,611	71	203,418	6,497	5.88	43
3005965	GTTCGTCCTTATGCA	*C. caesia*	138	515,265	409,195	290,680	71	217,487	6,659	6.81	42
2062291	AGCAATCTAATGCAG	*C. humilis*	84	1,690,664	1,209,446	504,923	42	179,477	6,710	10.91	42
2062251	GCTTCGGCTGTTGCA	*C. ovalifolia*	84	1,464,023	1,060,658	570,664	54	175,081	6,732	13.21	41
2062173	ATTGAACGCAATGCA	*P. altiplana*	84	1,606,363	1,091,915	934,182	86	330,246	11,297	11.61	2
2062180	AATCCATGGTCGTGC	*P. altiplana*	84	1,550,060	1,082,906	913,890	84	334,319	11,188	11.14	3
3006922	AGGAATAGGTTGCAG	*P. bajeensis*	138	502,998	351,699	307,235	87	340,039	10,564	4.68	8
3005861	CCACAGATGGACAGT	*P. bajeensis*	138	584,485	378,074	334,168	88	269,486	9,171	6.46	20
3402121	CTGTCGGTTAGTGCA	*P. bonjardinensis*	138	1,352,306	886,141	750,449	85	428,895	11,321	12.11	2
3402130	ACTGATTCTATAGTG	*P. bonjardinensis*	138	1,109,601	744,265	662,993	89	418,278	11,317	9.65	2
2986501	AGCCAACGGTCATGC	*P. mantiqueirensis*	138	508,948	282,720	238,691	84	242,039	9,155	4.84	20
2986502	GCGAGCCTACACGTG	*P. mantiqueirensis*	138	478,210	267,062	217,469	81	261,432	9,763	4.20	15
3005852	TCCACGTGGAGTGCA	*P. reitzii*	138	616,687	428,152	361,044	84	365,632	10,849	6.04	6
3005900	CGGTATATCATTCGT	*P. reitzii*	138	686,099	446,092	340,123	76	360,761	10,864	5.82	5
3005841	CGTTGACGTGTGCAG	*P. saxicola*	138	663,313	455,783	368,121	81	375,048	10,993	6.09	4
3005853	CGCAACGGCAGACGT	*P. saxicola*	138	624,327	427,737	372,216	87	366,637	10,975	6.72	5
2062327	GCCTGCGGTCTTGCA	*P. scheideana*	84	1,884,537	1,367,671	1,179,368	86	345,643	11,343	15.89	1
2062328	TTACCACTCATGCAG	*P. scheideana*	84	2,114,285	1,537,525	1,286,199	84	356,137	11,348	17.69	1
3005848	GTTCTCTTGTCGCGT	*P. integrifolia depauperata*	138	611,116	372,987	339,139	91	281,681	9,525	5.88	17
3005860	GCTGTGGACTGGAGT	*P. integrifolia depauperata*	138	626,133	401,560	366,404	91	335,941	10,678	6.12	7
3402162	TATTCTCAGCGATGC	*P. integrifolia integrifolia*	138	1,108,654	718,007	591,886	82	403,067	11,281	8.83	2
3402161	TCGCCGCTTCGTGCA	*P. integrifolia integrifolia*	138	1,386,933	856,910	762,826	89	407,846	11,294	9.27	2
3005838	GAGCCGCGGAACAGT	*P. correntina*	138	595,280	419,349	370,243	88	324,314	10,419	6.82	9
3005909	TGCGATGGTGTGCAG	*P. correntina*	138	651,673	436,559	403,536	92	331,738	10,441	5.97	9

3006917	TACGCAGGCGTTGCA	*P. inflata1*	138	412,523	280,128	228,041	81	278,336	9,230	3.78	20
3005837	ATGCCAATATTAGGT	*P. inflata1*	138	587,743	411,185	314,060	76	351,822	10,854	5.52	6
3402144	AGGTTGGCGGTATGC	*P. inflata2*	138	1,379,945	878,346	756,194	86	412,300	11,359	11.01	1
3402148	TTACTCCTTAGCGGT	*P. inflata2*	138	1,122,057	717,554	625,730	87	395,954	11,276	9.41	2
3005914	TGTGCGTTAGGAGAT	*P. interior1*	138	594,035	348,383	308,069	88	291,435	9,964	5.25	13
3005855	TTCACCGAATATTGC	*P. interior1*	138	534,488	358,995	311,741	87	331,962	10,400	5.78	10
3006928	CAGAATCTGGAGTGC	*P. interior2*	138	602,569	404,206	325,781	81	300,476	9,936	5.34	14
3006934	TTATATCAGCCGATG	*P. interior2*	138	421,631	299,216	247,001	83	235,523	7,840	5.75	32
2062295	GAATGGTTGCTTGCA	*P. guarapuavensis1*	84	1,688,198	1,247,969	1,058,955	85	343,004	11,355	12.65	1
2062296	AACCGCAGGATGCAG	*P. guarapuavensis1*	84	2,111,845	1,420,938	1,205,947	85	346,149	11,373	17.28	1
2062317	CTATACTGTGTTGCA	*P. guarapuavensis2*	84	1,703,332	1,216,121	783,305	64	335,740	11,337	11.27	1
2062318	CTATAGCACAGTTGC	*P. guarapuavensis2*	84	1,751,717	1,255,324	1,067,913	85	337,755	11,217	14.05	2
2062332	CATGGTTGTGATGCA	*P. guarapuavensis2*	84	1,749,299	1,210,621	880,828	73	335,712	11,371	12.17	1
2062334	GTTATCTGGCGTGCA	*P. guarapuavensis2*	84	1,928,956	1,413,428	1,206,914	85	350,259	11,392	16.02	1
3005872	ATAACAACCTGATTG	*P. secreta*	138	552,463	377,208	317,307	84	339,337	10,588	4.99	8
3006911	GAGTTGCCACACTTG	*P. secreta*	138	405,928	245,562	220,991	90	305,681	10,228	4.09	11
3005921	AGTAGCGAGGTATTG	*P. exserta*	138	582,800	381,838	361,283	95	318,220	10,203	4.91	11
3005885	CACGCTGGTAAGGTT	*P. exserta*	138	599,024	354,217	338,917	96	318,013	10,255	5.73	11
2062352	AGCGTAGACCGTGCA	*P. interior3*	84	1,954,475	1,265,682	1,078,937	85	339,505	11,367	15.16	1
2062161	CTGCTATTGAGTGCA	*P. interior3*	84	1,869,980	1,273,695	1,120,814	88	348,604	11,370	13.11	1
3005897	GCAAGTGTGGCACTT	*P. axillaris subandina*	138	531,731	343,893	323,768	94	324,188	10,339	4.77	10
2062349	ACGACTGCCAGCGTG	*P. axillaris subandina*	84	2,389,837	1,436,423	1,337,549	93	306,335	10,819	15.29	6
3005850	CCATGTGGCAGGATG	*P. toropiensis*	138	601,842	424,757	296,102	70	335,402	10,600	4.49	8
3005862	CCGTTCCGTAGATTG	*P. toropiensis*	138	544,709	386,107	287,449	74	326,392	10,307	5.21	10
3005920	GCCTTGCACGATCTT	*P. axillaris axillaris*	138	582,242	403,023	344,822	86	358,901	10,842	6.48	6

Table S2. Sampling information

Sequence ID	Species	Geographic coordinates	Voucher	BioSample accessions
3006023	*Calibrachoa spathulata*	-51.22/-26.44	ICN 160328	SAMN47304359
3005965	*Calibrachoa caesia*	-55.55/-27.25	BHCB143909	SAMN47304360
2062291	*Calibrachoa humilis*	-57.58/-29.24	BHCB143937	SAMN47304361
2062251	*Calibrachoa ovalifolia*	-52.89/-30.03	BHCB75137	SAMN47304362
2062173	*Petunia altiplana*	-49.60/-28.06	BHCB 96683	SAMN47304322
2062180	*Petunia altiplana*	-49.78/-28.20	BHCB 99752	SAMN47304323
3006922	*Petunia bajeensis*	-54.13/-31.40	BHCB 102127	SAMN47304324
3005861	*Petunia bajeensis*	-54.13/-31.40	BHCB 102127	SAMN47304325
3402121	*Petunia bonjardinensis*	-49.75/-28.26	BHCB 80098	SAMN47304326
3402130	*Petunia bonjardinensis*	-49.75/-28.26	BHCB 80098	SAMN47304327
2986501	*Petunia mantiqueirensis*	-45.88/-22.68	BHCB78268.	SAMN47304328
2986502	*Petunia mantiqueirensis*	-45.88/-22.68	BHCB 96683	SAMN47304329
3005852	*Petunia reitzii*	-49.48/-27.85	BHCB 80068	SAMN42638962
3005900	*Petunia reitzii*	-49.48/-27.85	BHCB 80068	SAMN42638954
3005841	*Petunia saxicola*	-49.73/-27.58	BHCB 80065	SAMN42638970
3005853	*Petunia saxicola*	-49.73/-27.58	BHCB 80065	SAMN42638971
2062327	*Petunia scheideana*	-49.30/-26.20	BHCB 80048	SAMN47304330
2062328	*Petunia scheideana*	-49.30/-26.20	BHCB 80048	SAMN47304331
3005848	*Petunia integrifolia depauperata*	-50.93/-31.12	NA	SAMN47304332
3005860	*Petunia integrifolia depauperata*	-52.16/-31.70	ICN 181859	SAMN47304333
3402162	*Petunia integrifolia integrifolia*	-54.08/-30.69	ICN 158636	SAMN47304334
3402161	*Petunia integrifolia integrifolia*	-55.94/-29.85	BHCB 140465	SAMN47304335
3005838	*Petunia correntina*	-58.87/-29.76	BHCB 201078	SAMN47304336
3005909	*Petunia correntina*	-58.87/-29.76	BHCB 201078	SAMN47304337
3006917	*Petunia inflata1*	-54.48/-28.15	BHCB 114603	SAMN47304338
3005837	*Petunia inflata1*	-54.63/-27.83	BHCB 114610	SAMN47304339
3402144	*Petunia inflata2*	-53.91/-27.40	BHCB 156818	SAMN47304340
3402148	*Petunia inflata2*	-53.91/-27.40	BHCB156818	SAMN47304341
3005914	*Petunia interior1*	-53.05/-27.62	BHCB 114615	SAMN42638984
3005855	*Petunia interior1*	-52.30/-26.57	BHCB 114616	SAMN42638980
3006928	*Petunia interior2*	-52.03/-28.93	BHCB 114596	SAMN42638988
3006934	*Petunia interior2*	-52.03/-28.93	BHCB 114596	SAMN42638987
2062295	*Petunia guarapuavensis1*	-51.21/-25.33	BHCB 96577	SAMN47304342
2062296	*Petunia guarapuavensis1*	-51.21/-25.33	BHCB 96577	SAMN47304343
2062317	*Petunia guarapuavensis2*	-51.48/-26.28	BHCB 96623	SAMN47304344
2062318	*Petunia guarapuavensis2*	-51.15/-26.53	BHCB 96642	SAMN47304345
2062332	*Petunia guarapuavensis2*	-51.04/-26.74	BHCB 15096	SAMN47304346
2062334	*Petunia guarapuavensis2*	-51.04/-26.74	BHCB 15096	SAMN47304347
3005872	*Petunia secreta*	-53.55/-30.54	ICN181341	SAMN47304348
3006911	*Petunia secreta*	53.28/30.21	ICN181342	SAMN47304349
3005921	*Petunia exserta*	-53.49/-31.22	BHCB140448	SAMN47304350
3005885	*Petunia exserta*	-53.50/-30.83	BHCB79896	SAMN47304351

2062352	*Petunia interior*	-54.27/-28.34	BHCB 114601	SAMN47304352
2062161	*Petunia interior*	-54.27/-28.34	BHCB 114601	SAMN47304353
3005897	*Petunia axillaris subandina*	-64.52/-30.85	ICN 164575	SAMN47304354
2062349	*Petunia axillaris subandina*	-64.93/-31.75	BHCB 140429	SAMN47304355
3005850	*Petunia toropiensis*	-54.08/-29.45	BHCB206043	SAMN47304356
3005862	*Petunia toropiensis*	-54.08/-29.45	BHCB206043	SAMN47304357
3005920	*Petunia axillaris axillaris*	53.31/-31.31	ICN158639	SAMN47304358
